# The adequacy of measures of gender roles attitudes: a review of current measures in omnibus surveys

**DOI:** 10.1007/s11135-017-0491-x

**Published:** 2017-03-03

**Authors:** Jessica Gabriele Walter

**Affiliations:** GESIS- Leibniz Institute for the Social Sciences, B2,1, P.O. Box 122155, 68072 Mannheim, Germany

**Keywords:** Gender role attitudes, Measure, Longitudinal analysis, Omnibus surveys

## Abstract

The measures of attitudes toward gender roles included in many representative international and national omnibus surveys were developed mostly in the 1970s and 1980s with a focus on the male breadwinner model. This article deals with the issue of whether the measures provided in these omnibus surveys need to be adjusted to specific social changes. A review of these measures has found that adjustments have occurred in a limited way that focused on the role of women and disregarded the role of men. Furthermore, most of these measures only examined the traditional roles of men and women. More egalitarian role models have not been considered sufficiently. In addition, most items that have been measured are phrased in a general form and, for example, do not specify parents’ employment or the ages of children. A specification of these aspects of measurement would help to clarify the conceptual meaning of the results and increase the possibility of more accurately analyzing gender role attitudes over time.

## Introduction

Beliefs about the appropriate roles for men and women regarding the division of paid labor, homework, and childcare often are referred to as *gender role attitudes* or as *gender ideology* (e.g., Davis and Greenstein 2009). Several quantitative studies (e.g., Bolzendahl and Myers 2004; Brewster and Padavic 2000; Cotter et al. 2011; Lee et al. 2007) that have examined the change of these attitudes since the end of the 1970s have shown that traditional gender role attitudes have declined, and egalitarian attitudes have increased. Even if gender role attitudes differ between various groups in a society with the, “well educated, the less religious, the unmarried, and [… the] postmaterialists” (Inglehart and Norris 2003, p. 47) tending to be more egalitarian, Inglehart and Norris (2003) have shown that differences are larger between societies than between groups within a society. Clear evidence exists that the shift to an industrial and an even more influential postindustrial society leads to more egalitarian gender role attitudes. Thus, most studies concerned in some way with gender role attitudes rely on the measures provided in surveys. However, to evaluate change in gender roles attitudes over time, we must critically reflect on the measures used to carry out this evaluation. Only by measuring the underlying construct in the same way over time will our observations about changing attitudes be valid. However, if the interpretation of these measures changes, we may need to consider adjusting the measures themselves. In other words, the validity of measures in use has to be evaluated in light of their context. This scenario especially applies if the same measures are used over a long period of time, and social change is likely to occur or does occur. Social change also may lead to new aspects of gender roles that have not been considered adequately using the old measures.

This article analyzes whether social change leads to a necessity to adjust measures of gender role attitudes, and also assesses the validity of the measures in use. I begin by describing the concept of *gender role attitudes* and why observing change might be a problem. After a description of relevant societal developments, I examine how they affect measures of gender role attitudes. Therefore, I systematically review the measures used in selected national and international omnibus surveys. Finally, I summarize the results and discuss necessary considerations for future studies of gender role attitudes and beyond.

## Concept of gender role attitudes

Regarding gender roles, I focus on the measures concerning “the assignment of different adult social responsibilities to men and women” (Pleck 1977, p. 182), which are used to measure the attitudes about the appropriate roles of men and women. To better understand these attitudes and evaluate the need for improvement, we need to conceptualize them. However, so far, no generally accepted concept has been agreed on for use. Therefore, a concept needs to be developed that includes attitudes about gender roles. The main distinction regarding gender roles has been drawn between the roles ascribed to the public sphere and the roles ascribed to the private sphere. The public sphere roles are related to community or public office (e.g., party executive or president) (e.g., Baber and Tucker 2006) and to occupations (e.g., taxi driver or secretary). The private sphere roles usually are related to a distinction between roles in a partnership and those concerning parenthood (e.g., Baber and Tucker 2006). Furthermore, the intersection of these two spheres is important (see Scott 2010). Another distinction can be drawn between attitudes towards role ascription and attitudes towards role conflict. *Attitudes towards role ascription* are about to which roles a man or a woman should conform. In other words, study respondents ascribed a role to a man or a woman (e.g., “a woman has to have children in order to be fulfilled” European Values Study 1981). *Attitudes towards role conflict* address how these conflicts—for example, which can occur between the public and private spheres—are evaluated. Conflicts also can occur within spheres, for example, by neglecting a partner to spend time with the children. Finally, attitudes broach a *segregation of roles*—how couples should divide the roles of the private and public spheres within a relationship. An example is: “A man’s job is to earn money; a woman’s job is to look after the home and family” (British Social Attitudes Survey 1984). Therefore, the distinction between role ascription, role conflict, and role segregation approximates a distinction made by Funk (1991) between role segregation, role combination, and role conflict.

**Fig. 1:**
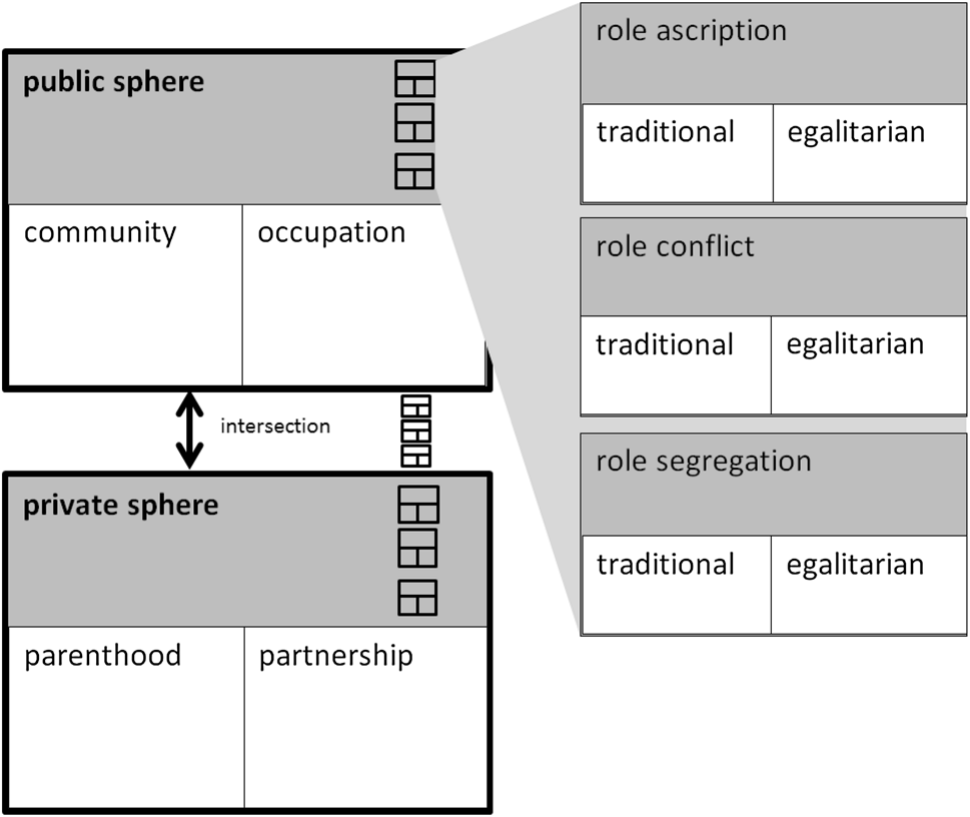
The concept of *gender role attitudes*

With respect to these different aspects, roles can be allotted to a traditional or an egalitarian point of view. The former implies that the private sphere, for example, is assigned to women, and they are restricted to complying with their family responsibilities. In contrast, egalitarian attitudes are demonstrated, for example, when someone believes that men and women should share equally the responsibility for family tasks and that women as well as men should participate in paid work. The distinction between traditional and egalitarian roles can be made regarding the different aspects of gender roles: role ascription, role conflict, and role segregation. Figure 1 presents an outline of the concept of *gender role attitudes*.

Theoretically, we can distinguish nine different aspects of gender role attitudes:

- Role ascription within the public sphere.
- Role ascription within the private sphere.
- Role ascription at the intersection of these two spheres.
- Role conflict within the public sphere.
- Role conflict within the private sphere.
- Role conflict at the intersection of the two spheres.
- Role segregation regarding the public sphere.
- Role segregation regarding the private sphere.
- Role segregation at the intersection of the two spheres.

The concept of *gender role attitudes* aims to provide the possibility of allocating the measures of gender role attitudes developed so far. Based on this concept, measures of gender role attitudes can be evaluated with respect to their coverage and gaps. For the development of measures, however, the aspect of observing change also must be considered.

## Measures of gender roles over time

The evaluation of attitude change by Smith (2005) reminds us that we need to consider how change is measured. According to Smith’s first law, we can only measure change if measures are not changed over time. However, constant measures may produce non-constant measurement (Smith’s second law), which makes it necessary to change the measures. The first law refers to the problem that even small changes in measures may change the measurement and lead to invalid interpretations of attitude change. In this case, a change in attitudes may be attributed only to changes in measures. The second law refers to the possibility that the functional equivalence of measures is not given due to the changes in the substantial meaning associated with them or of their applicability, which can be caused by societal developments. As a consequence, we must violate the first law and change the measures to measure the same concepts over time.

Braun (2009) has stated that the functional equivalence of measures is only a given if their interpretation does not change. While this statement refers to cross-cultural comparisons, the same applies for cross-temporal comparisons. Thus, the interpretation is contingent on the personal experiences of the respondent—her/his socialization and personal living conditions, such as the employment of his/her mother, their own employment, and family status—, the cultural context, and the context of questions in the questionnaire (c.p. Braun 2006; Tfaily 2010). The cultural context frames gender roles by legal regulations or creates the norms for gender roles in society. Societal developments can affect the interpretation of measures by changing personal experiences and the cultural context. Barth (2016) has shown that for Britain, for example, gender role attitudes have become more complex due to social change. Regarding the measures of gender role attitudes, a large diversity of potential influential context variables exists. Next, I describe which societal developments may affect the measures of gender role attitudes. Thus, I focus on the developments in countries—USA, Germany, Japan, Italy, Sweden, and UK—in which the measures of gender role attitudes also represent different cultural contexts, for example, regarding female participation in the labor force.

## Societal developments affecting measures of gender role attitudes

Most measures of gender role attitudes were developed in the late 1970s and 1980s when the dominant model for living together was the male bread-winner model. This family model was widespread with some variation in Western countries (Crompton 1999).

### Developments in education and labor force participation

One of the developments that led to a decline of the male bread-winner model was more women becoming better educated. Today, women invest more in education than they used to in the late 1970s and 1980s (Becker et al. 2010). The increase in women’s education also is connected to female labor force participation (e.g., Jaumotte 2004). In 1970, the female labor force participation rate was about 50% in the USA, Germany, and Japan; about 60% in Sweden; and about 30% in Italy, which was rather low. Since then, female labor force participation has increased in these countries (OECD 2014c). At the same time, only small changes can be observed with respect to the male employment rate of approximately 90%. Further changes in female participation were changes in working hours and the participation of mothers. On the one hand, the part-time work of women increased in Germany, Japan, and Italy; decreased in Sweden; and stayed approximately the same in the USA and the UK (OECD 2014b). On the other hand, a look at the labor force participation rate of mothers shows that it increased since the 1970s, although mothers with younger children are still less likely to work for pay and often work part-time (Peuckert 2012; Macran et al. 1996; OECD 2014d; Mosisa and Hipple 2006). Changes in employment also mean that the male bread-winner model (for example, in 1990 34% of households with couples in West Germany fit this model, but in 2007, only 20% fit) is being replaced increasingly by a model in which the male partner works full-time, and the female partner works part-time (in 1990 26% and in 2007 40%) (Peuckert 2012; see also McCulloch and Dex 2001; OECD 2014e). Hence, while the women is more likely to “win bread” as well, she usually is not employed to the same extent as her partner (OECD 2014a). In summary, female labor force participation has changed insofar as it has increased and become more differentiated regarding working hours and regarding women with or without children, depending on the age of the children. Developments in labor force participation and education are accompanied by the following developments in family structure.

### Developments in family structure

**Fig. 2:**
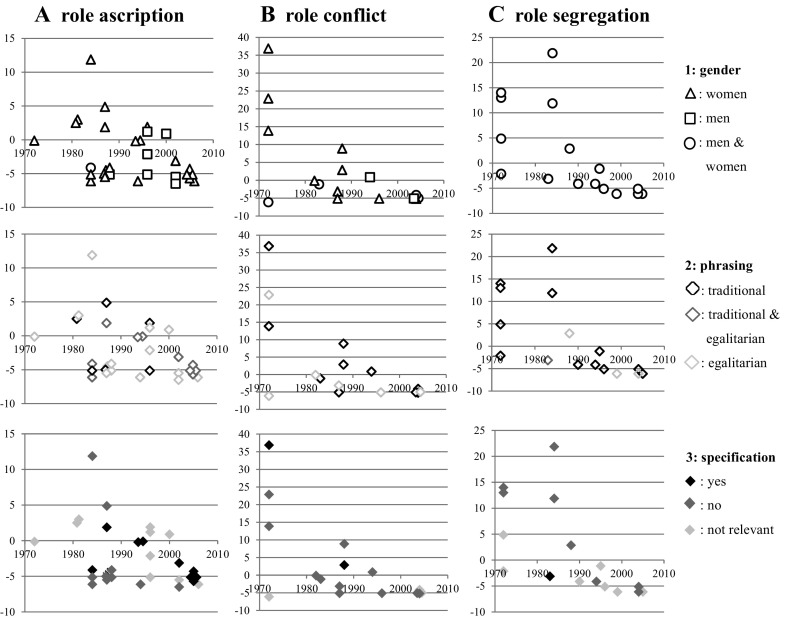
Measures of gender role attitudes. *Note* x-axis: first year of measurement of item; y-axis: number of years item is conducted in relation to mean number of years conducted of all items

Many changes in family structure can be observed since the 1970s. In that decade, marriage was more common than today and women married at younger ages (OECD 2014h; Peuckert 2012; Statistics Bureau Japan 2013; Elliott et al. 2012). Furthermore, the first child was born much earlier than today, and the fertility rate was higher (OECD 2013, 2014f). Alternative living arrangements to the married heterosexual couple were not widespread. Few children were born non-marital, and cohabitation was not common (OECD 2011). Since the 1970s, cohabitation is widely practiced, especially among younger people (OECD 2014i; Nazio and Blossfeld 2003). Additionally, more and more women remain unmarried and divorce rates have risen (see Peuckert 2012, p. 305 ff; Statistics Bureau Japan 2013; OECD 2014h; Elliott et al. 2012; Fig. 2). In other words, families are not based necessarily on a married couple any more. Also, the rising number of single parents has consolidated this fact (OECD 2011). Another development that has affected family is the rising number of childless women (Peuckert 2012; OECD 2014g). In addition, in many countries legal changes also have occurred, which have encouraged males to take a greater share in the child-rearing of small children by being offered paid parental leave. However, many men do not use this opportunity as much as they could. Due to the increasing number of working women, the pressure on men has probably increased with respect to their participating more in housework and child-rearing (e.g., Breen and Cooke 2005), and men also have to balance work and family somehow (Ranson 2001).

The previously described changes in female education and labor force participation, as well as changes in family structure occurred in many Western countries in similar ways, although there were and are still are differences between countries. For example, Sweden has a higher participation of women in the labor force than Italy, and the change in working hours for women is less pronounced in the USA or UK (OECD 2014a, c). Despite country-specific developments, family formation and living arrangements have become more differentiated, and the participation of women in the labor force has increased in Western countries over time.

### Effects on measures of gender role attitudes

Why should the previously described developments affect measures of gender role attitudes? According to exposure-based or interest-based explanations, socialization theories, and control models, the personal experience of a person is influenced among other things by the family situation and labor force participation. Relying on exposure-based explanations (Bolzendahl and Myers 2004), we would assume that the personal experience of a person affects how he/she evaluates gender roles. Individuals in the 1970s probably had personal experiences that were different from individuals living today in 2017. It was less likely that mothers worked. Deviation from the male bread-winner model based on a married couple was not widespread. For women, it was less likely to gain a higher education and thereby be exposed to non-traditional roles of men and women. According to socialization theories, which also are based on the idea of the influence of exposure, children learn about roles by imitating their parents’ gender roles (Myers and Booth 2002). Several studies have found, for example, that if a mother is employed, their children have more egalitarian gender role attitudes (Davis and Greenstein 2009; Boehnke 2011). Interest-based explanations refer to personal experience and to the influence of personal living circumstances on how someone perceives the roles of men and women in society by adjusting them to their own interests (Bolzendahl and Myers 2004; Kroska and Elman 2009; Corrigall and Konrad 2007; Abe 2011) with gender role attitudes also being affected, for example, by the participation of women in the labor force (Cunningham 2008). Control models also assume that people possibly adapt their attitudes to their own living circumstances to avoid cognitive discrepancies between their own attitudes and living circumstances (Kroska and Elman 2009).

In addition to personal experiences, the measures of gender role attitudes may also be affected by the cultural context in which someone lives. This context has changed insofar as societies in general are less traditional nowadays than in the 1970s. Women’s independence has become a part of the modernization process in societies (c.p. Inglehart and Norris 2003). In post-industrial societies, most women do not accept traditional roles anymore, and gender equality has become an important issue in political debate (Inglehart and Welzel 2005). Gender roles have converged in postindustrial societies “because of a structural revolution in the paid labor force, in educational opportunities for women, and in the characteristics of modern families” (Inglehart and Norris 2003). In other words, gender equality has become a new societal norm, and persons who support traditional gender role attitudes are said to deviate from this norm and may face negative consequences. The acceptance of female participation in the labor force also may be influenced by increasing divorce rates and a rising number of single parents, which may increase the need for women to become financially independent. Also, the decreasing number of children born should have an effect on how gender roles are evaluated, since fewer children are affected by how their parents divide the roles of the private and public spheres.

Another development that also facilitates a more egalitarian opinion regarding gender roles is increasing secularization. With a shift away from church membership, and its related traditional thinking, towards more individualistic religious beliefs, a shift toward less traditional gender role attitudes also can be observed.

The question is whether these societal developments have led to a true change of gender role attitudes or whether we only observe these changes due to an adaptation of socially desirable behavior to new social norms. In the latter case, the change we observe results from the socially desirable behavior of respondents insofar as they think that the expression of more egalitarian attitudes is desirable. By expressing socially desirable behavior, respondents can avoid potential negative reactions from their environment. A shift to a social norm that favors egalitarian models of the division of labor should result in an increase in the expression of egalitarian attitudes. Unfortunately, it is almost impossible to assess the true gender role attitudes of respondents. In addition, social desirability is difficult to measure. Even if measures to assess the tendency of a respondent to respond in a socially desirable manner (e.g., Crowne–Marlowe need for approval scale) are available, most surveys do not include them. With respect to the assessment of gender role attitudes, we do not know to what extent they are influenced by social desirability. A high proportion of “don’t know” answers or refusals indicates that questions are prone for social desirability (Krumpal 2013). Measures of gender role attitudes, however, do not show a high proportion of item non-response or “don’t know” answers as the example of the German General Social Survey (GGSS) shows. Studies also have indicated that social desirability differs by mode (Kreuter et al. 2008). A first comparison of a web-pretest for the GGSS with the original survey distribution (conducted face-to-face) has indicated that gender role attitudes do not differ in systematic ways, and that these attitudes have not differed to a large extent between the pretest and the survey in 2012. This finding also attenuates earlier findings that the sex of the interviewer influences gender role attitudes (Kane and Macaulay 1993) because in web surveys, the sex of the interviewer could not possibly influence the behavior of respondents. Furthermore, we can observe changes in behavior as well as in attitudes: fathers invest more time in childcare, parental leave policies have changed (Akgunduz and Plantenga 2013; Boll et al. 2014; Goldscheider et al. 2015), and female participation in the labor force has increased. Social desirability can explain only some of these changes, which supports the assumption of a real change in gender role attitudes. However, future studies should examine in detail the extent to which gender role attitudes are influenced by social desirability.

Social changes suggest that more people have been exposed to working women, and especially working mothers, and additionally, to the idea that the employment of women does not necessarily equate with full-time work. This exposure should affect the acceptance of female participation in the labor force. Since personal experience and the cultural context both have changed over time, the question arises as to whether and how measures of gender role attitudes apply to these changes. Questions come into focus, such as how the part-time employment of women is evaluated or how the role of men in the household is perceived. Can we evaluate such attitudes with the measures in use? The following section reviews measures of gender role attitudes and evaluates to what extent they adapt to the previously described changes.

## Review of measures of gender role attitudes

The measures of gender role attitudes included in almost all national or international omnibus surveys always cover several aspects of the *gender roles* concept. However, these surveys seldom cover all aspects of gender roles, and often include shorter scales of gender role attitudes than psychological measures, such as the Attitudes Toward Women Scale (AWS) or the Social Roles Questionnaire (see Baber and Tucker 2006; McHugh and Frieze 1997). An explanation for these shorter scales is probably the time constraints of the survey.

The following paragraphs introduce the measures used in international, European, and national cross-sectional omnibus surveys (exception being the longitudinal Generations and Gender Programme) with a large sample representative of the national population. The focus is on omnibus surveys in which attitudes about gender roles are asked in short scales, which can be analyzed together with a number of background variables and other topics. These surveys are the USA General Social Survey (GSS; 1972—ongoing; 1972–1994 ~annual; since 1994 biannual; ~N 1500–4500), the German General Social Survey (GGSS/ALLBUS; 1982—ongoing; biannual; ~N 3500), the British Social Attitudes Survey (BSA; 1983—ongoing; annual; ~N 3000), the Japanese General Social Survey (JGSS; 2000—ongoing; 9 waves; ~N 2000–5000), the Taiwan Social Change Survey (TSCS; 1984—ongoing; 6 rounds; ~N 1100–4300), the Australian Survey of Social Attitudes (AuSSA; 2003—ongoing; 7 waves; ~N 1500–8000), the Korean General Social Survey (KGSS; 2003—ongoing; annual; ~N 1300–1600), and the East Asian Social Survey (EASS; 2003—ongoing; 4 waves; ~N 2500–8000 each country). The considered European and international omnibus surveys are the European Social Survey (ESS; 2002—ongoing; biannual; ~N 800–1500 each country), the European Values Study (EVS; 1981—ongoing; 4 waves; ~N 1500 each country), and the World Values Survey (WVS; 1981—ongoing; 6 waves; ~N 1000–2000 each country). Finally, the measures used in the Generations and Gender Programme (GGP; 2004—ongoing; 3 waves; ~N 9000 each country) and the International Social Survey Programme (ISSP; 1984—ongoing; annual; ~N 1500 each country) are reviewed. The GGP is an international survey with a focus on gender, and the ISSP focuses regularly on family and gender as topic (repeated four times so far). A study by Davis and Greenstein (2009) with a focus on surveys in the USA such as the National Longitudinal Survey of Youth and the National Study of the Changing Workforce is therefore supplemented. Although the considered surveys comprise a good range of different cultural contexts, the selection does not claim to cover all national omnibus surveys with a representative sample and large sample size.

Starting in 1972, the GSS was one of the first surveys to include measures of gender role attitudes and other general social surveys and international surveys followed (e.g., BSA, ALLBUS, EVS, ISSP, JGSS, WVS). Table 1 provides an overview of the measures of gender role attitudes, which are asked more than once (in more than one survey or in more than one round of the same survey). Thus, an analysis over time or cross-culturally is possible. A measure of the actual behavior of respondents regarding gender roles and concepts used in androgyny like masculinity and femininity, which focus more strongly on differences in personality between men and women, are not included . Table 1 is based on the documented English translations of the surveys and on item databases provided for individual surveys. Items are chosen by a semantic analysis. Questionnaires or item databases were searched for words related to gender and family, such as *woman, husband, wife*, and *children*, and items were selected when they described gender roles. Table 1 is organized according to the nine conceptual aspects of gender roles presented in Fig. 1 and provides information about the phrasing of items. Items of different surveys with similar phrasing were counted as the same item. Additionally, it provides information about the first and last year of measurement for all the surveys. The number of surveys or rounds in a survey that included gender role attitudes also is reported.

**Table 1:** Measures of gender role attitudes in national and international omnibus surveys

Item	First/last year	Asked in survey^frequency of item in respective survey over time^
*(a) Ascription public sphere*		
(1) Having a job is the best way for a **woman** to be an independent person^a^	1984/2012	BSA^5^, JGSS^2^, TSCS^4^, EVS^3^, WVS^2^, ISSP^3^, KGSS^1^
(2) Which of these best describes the reasons why many **married women** work (1) for the company of other people; (2) need money for basic essentials; (3) to earn money of their own; (4) to earn money to buy extras; (5) to follow a career; (6) work is a change; (7) working is the normal thing to do	1984/1991	BSA^2^
(3) Do you think that the job is particularly suitable for **men** only, particularly suitable for **women** only, or suitable for both equally…(1) bus driver; (2) computer programmer; (3) airline pilot; (4) bank manager; (5) car mechanic; (6) director of an international company; (7) family doctor/GP; (8) local councilor; (9) member of Parliament; (10) nurse; (11) police officer; (12) secretary; (13) social worker	1984/1994	BSA^4^
(4) **Married women** have a right to work if they want to, whatever their family situation	1987/1994	BSA^3^
(5) Do you agree or disagree that a **woman** becomes the Empress?	2006/2012	JGSS^2^
(6) If your party nominated a **woman** for President, would you vote for her if she were qualified for the job?	1972/2010	GSS^8^
(7) Most **women** have to work these days to support their families	1994/2003	ISSP^1^, KGSS^1^
*(b) ascription private sphere*		
(1) **Men** should cook and look after themselves	2000/2010	JGSS^9^
(2) **Men** ought to do a larger share of household work than they do now	2002/2006	EASS^1^, ISSP^1^
(3) **Men** ought to do a larger share of child care than they do now/A **father** should be as heavily involved in the care of his children as the mother	2002/2003	AuSSA^1^, ISSP^1^
(4) The authority of **father** in a family should be respected under any circumstances/The **husband** is the head of the household and the wife should be obedient to him	1996/2008	TSCS^1^, EASS^2^
(5) A **woman** can have a child as a single parent even if she doesn’t want to have a stable relationship with a man^a^	1981/2012	EVS^4^, GGP^3^, WVS^4^
(6) A **woman** has to have children in order to be fulfilled/Do you think that a woman has to have children in order to be fulfilled or is this not necessary?/Women must raise children to have a fulfilled live	1981/2012	EVS^4^, GGP^3^, WVS^3^, TSCS^1^
(7) **Married women** are generally happier than unmarried women/Without a doubt, a **woman’s** happiness lies in a marriage	1996/2012	EASS^2^, JGSS^8^
(8) **Men** can have a fulfilling life without children/Men do not have to raise children to have a fulfilled life	1996/2012	EASS^1^, EVS^2^, GGP^3^
(9) **Men** can have a fulfilling life without marriage/Men can still have a fulfilled life without getting married	1996/2012	EASS^2^, JGSS^8^
(10) It’s mainly the **mother**’s responsibility to discipline the children	1984/2000	TSCS^3^
*(c) Ascription private and public sphere*		
(1) A **single father** can bring up his child as well as a married couple^a^	1988/1996	ISSP^1^, TSCS^2^
(2) A **single mother** can bring up her child as well as a married couple^a^	1988/2012	GSS^1^, ISSP^1^, TSCS^2^
(3) A job is alright, but what most **women** really want is home and children	1987/2012	BSA^4^, EVS^3^, ISSP^4^, KGSS^1^, WVS^1^
(4) How much do you agree or disagree that **women** shouldn’t try to combine a career and children	1987/1994	BSA^3^
(5) How much do you agree or disagree that if children are well looked after, it’s good for a **woman** to work	1987/1994	BSA^3^
(6) Do you think that **women** should work outside the home full-time, part-time or not at all under these circumstances (1) after marrying and before there are children; (2) when there is a child under school age; (3) After the children leave home; (4) when a couple has not yet had a child; (5) After the youngest child starts school (6) After all children complete elementary school^b^	1987/2012	BSA^4^, ISSP^4^, TSCS^2^
(7) About the government ensuring that affordable, good quality child care was available. Thinking about a **single mother** when her child reaches school age. Which comes closes to your view about what the single mother should do…she has a special duty to go out to work to support her child//She has a special duty to stay at home to look after her child^a^	1994/2009	BSA^8^
(8) About the government ensuring that affordable, good quality child care was available. Thinking about a **single mother** with a child under school age. Which comes closes to your view about what the single mother should do…she has a special duty to go out to work to support her child//She has a special duty to stay at home to look after her child^a^	2005/2009	BSA^3^
(9) About a **single mother** with a child under school age. Which comes closest to your view? She has a special duty to go out to work to support her child//She has a special duty to stay at home to look after her child	1994/2009	BSA^8^
(10) About a **single mother** with a child of school age. Which comes closest to your view? She has a special duty to go out to work to support her child//She has a special duty to stay at home to look after her child	2005/2009	BSA^3^
(11) About a **married mother** with a child of school age. Suppose the government ensured that affordable, good quality child care was available. Which comes closest to your view? She has a special duty to go out to work to support her child//She has a special duty to stay at home to look after her child	2005/2009	BSA^3^
(12) About a **married mother** with a child under school age. Suppose the government ensured that affordable, good quality child care was available. Which comes closest to your view? She has a special duty to go out to work to support her child//She has a special duty to stay at home to look after her child	2005/2009	BSA^3^
(13) About a **married mother** with a child under school age. Which comes closest to your view? She has a special duty to go out to work to support her child//She has a special duty to stay at home to look after her child	2002/2009	BSA^5^
(14) About a **married mother** with a **child of school age. Which** comes closest to your view? She has a special duty to go out to work to support her child//She has a special duty to stay at home to look after her child	2005/2009	BSA^3^
*(d) Role conflict public sphere*		No items observed
*(e) Role conflict private sphere*		
(1) A **wife** should avoid earning more than her **husband** does/If a **woman** earns more than her **partner**, it is not good for the relationship/If a woman earns more money than her husband, it’s almost certain to cause problems/It is better if the husband’s income is higher than the wife’s	1983/2010	BSA^1^, GGP^3^, TSCS^1^, WVS^2^
(2) If the **husband** in a family wants children but the **wife** decides that she does not want any children, is it all right for the wife to refuse to have children?	1972/1996	GSS^2^
(3) **Women** should be able to decide how to spend the money they earn without having to ask their **partner**’s permission	2004/2012	GGP^3^
(4) The **husband** should be older than his **wife** ^a^	2004/2012	EASS^1^, GGP^3^, TSCS^2^
*(f) Role conflict private and public sphere*		
(1) A working **mother** can establish just as warm and secure a relationship with her children as a mother who doesn’t work^a^	1972/2012	ALLBUS^8^, BSA^2^, EVS^3^, GSS^8^, ISSP^4^, JGSS^2^, KGSS^1^, WVS^3^
(2) A preschool child is likely to suffer if his or her **mother** works^a^	1972/2012	ALLBUS^8^, AuSSA^2^, BSA^3^, EVS^3^, GGP^3^, GSS^8^, ISSP^4^, JGSS^9^, TSCS^4^, WVS^1^
(3) A child actually benefits if his or her **mother** has a job rather than just concentrating on the home	1982/2012	ALLBUS^8^
(4) A **woman** and her family will all be happier if she goes out to work	1987/1994	BSA^4^, ISSP^1^
(5) All in all, family life suffers when the **woman** has a full-time job^a^	1988/2012	BSA^2^, ISSP^5^, TSCS^4^
(6) A **woman** should be prepared to cut down on her paid work for the sake of her family	2004/2010	ESS^3^
(7) Family life often suffers because **men** concentrate too much on their work	1994/2012	GSS^7^, ISSP^1^, KGSS^1^
(8) Being a **housewife** is just as fulfilling as working for pay^a^	1988/2012	BSA^1,^EVS^3^, GGP^3^, ISSP^4^, KGSS^1^, WVS^5^
(9) It is more important for a **wife** to help her **husband**’s career than to have one herself^a^	1972/2012	ALLBUS^8^, EASS^2^, GSS^5^, JGSS^7^
(10) How much do you agree or disagree that if a **woman** takes several years off to look after her children it’s only fair her career should suffer.	1987/1994	BSA^3^
(11) It is more fulfilling for **women** to work for pay than to be a homemaker	1996/2001	TSCS^3^
(12) Children often suffer because their **fathers** concentrate too much on their work	2004/2012	GGP^3^
*(g) Role segregation public sphere*		
(1) **Women** should take care of running their homes and leave running the country up to **men**	1972/1998	GSS^6^
(2) Tell me if you agree or disagree with this statement: Most **men** are better suited emotionally for politics than are most **women** ^a^	1972/2012	GSS^13^
(3) On the whole, **men** make better political leaders than **women** do	1995/2012	GGP^3^, WVS^4^
(4) Politics is a **men’s** game, it is better for **women** not to be involved	1990/2012	TSCS^4^
(5) On the whole, **men** make better business executives than **women** do	2005/2010	WVS^2^
*(h) Role segregation private sphere*		
(1) **Men** should take as much responsibility as women for the home and children^a^	2004/2008	ESS^1^, EVS^1^
(2) Who do you think should do this—mainly the **man**, mainly the **woman**, or should the task be shared equally…(1) look after children when they are sick; (2) teach children discipline; (3) household shopping; (4) make the evening meals; (5) organize the household money and payment of bills; (6) repair the household equipment; (7) the evening dishes; (8) the household cleaning; (9) the washing and ironing/Which one of the following do you think is a fair way for a couple to share household work? Both should do half of the household work/Any method, as long as the couple reaches an agreement/Household work should be assigned according to each spouse’s expertise/skill or preference/Other	1983/2011	BSA^4^, TSCS^1^
(3) **Women** are more suitable for taking care of the family than **men** ^a^	1996/2008	EASS^1^, TSCS^2^
(4) In general, **fathers** are as well suited to look after their children as **mothers**	1999/2008	EVS^2^
(5) If parents’ divorce it is better for the child to stay with the **mother** than with the **father**	2004/2012	GGP^3^
*(i) Role segregation private and public sphere*		
(1) It is much better for everyone involved if the **man** is the achiever outside the home and the **woman** takes care of the home and family^a^/It’s better for a **husband** to take care of external matters, while a **wife** takes care of domestic matters/Families are more harmonious when the **husband** is in charge of the “external” affairs and the **wife** takes care of the “internal” affairs	1972/2012	ALLBUS^8^, GSS^12^, TSCS^2^
(2) A **man’s** job is to earn money; a **woman’s** job is to look after the home and family^a^/For a married couple, the **husband** should be in charge of the “external” affairs, while the **wife** takes care of the “internal” affairs	1984/2012	AuSSA^1^, BSA^6^, EASS^3^, ISSP^7^, JGSS^7^, KGSS^1^, TSCS^6^
(3) Do you approve or disapprove of a **married woman** earning money in business or industry if she has a **husband** capable of supporting her?^a^	1972/2012	ALLBUS^8^, GSS^5^, JGSS^9^
(4) When jobs are scarce, **men** should have more right to a job than **women**/During economic recession, it is all right for **women** to be laid-off before **men**/In times of high unemployment **married women** should stay at home	1984/2012	BSA^4^, EASS^1^, ESS^3^, EVS^3^,GGP^3^, TSCS^1^, WVS^5^
(5) It is not good if the **man** stays at home and cares for the **children** and the **woman** goes out to work	1994/2004	BSA^2^, ISSP^1^,KGSS^1^
(6) Both the **husband** and the **wife** should contribute to the household income^a^	1988/2012	EVS^3^, ISSP^4^, KGSS^1^, WVS^3^

*GSS* General Social Survey, *ALLBUS* German General Social Survey (GGSS), *BSA* British Social Attitudes Survey, *JGSS* Japanese General Social Survey, *AuSSA* Australian Survey of Social Attitudes, *TSCS* Taiwan Social Change Survey, *EASS* East Asian Social Survey, *GGP* Generations and Gender Programme, *ESS* European Social Survey, *EVS* European Values Study, *WVS* World Values Survey, *ISSP* International Social Survey Programme

^a^The item phrasing of some items differs between the different surveys

^b^Some items are asked only partly in the instrument; the EASS comprises TSCS, KGSS and partly the JGSS as well as the Chinese General Social Survey (no additional information available); The items conducted in the ISSP in the relevant survey years are integrated in the investigations of the GSS, BSA, and partly TSCS and KGSS and are only counted for the ISSP

The measures of gender role attitudes mainly were supposed to identify whether persons have a traditional point of view regarding gender roles or not. However, even if we know that someone refuses a traditional point of view, we do not necessarily have information about how egalitarian she/he is (Braun 2008) or the other way around. Since the division of labor between men and women can express itself in different forms on a continuum between traditional and egalitarian attitudes, some surveys, over time, have introduced more measures that refer to a more egalitarian role model. In addition to the distinction between egalitarian and traditional measures of gender role attitudes, we also need to look at the phrasing of the measurement items, since they differ regarding their degree of specification. They can be phrased in a general form (e.g., “Most women have to work these days to support their families,” e.g., ISSP) or be more specific (e.g., “All in all, family life suffers when the woman has a full-time job,” e.g., BSA). Empirical studies often show only two aspects of gender roles, which refer to role segregation and the consequences of employment (related to role conflict) (Braun and Scott 2009; Lee et al. 2007). These measures are worded mainly as statements that respondents evaluate using an agreement scale (2, 4, or 5 point scales).

Many measures of gender role attitudes aim to enable analyses over time. To evaluate whether and how measures account for societal developments, the items presented in Table 1 are systematically explored according to the following: (1) Does the item focus on the roles of men or women (or both)? (2) Is the focus of the item on egalitarian or traditional attitudes (or both)? and (3) Does the item specify the amount of employment or the ages of the children? The first aspect is important, since more women live non-traditional roles that also affect the role of men. An analysis by gender indicates whether measures consider gender by dealing with, for example, the role of men. The second aspect is important, since the division of labor became more differentiated over time, both for women and men. Items with a stronger egalitarian stance gain importance, since the non-traditional models of the division of labor within the family have become more important. The third aspect is relevant, since developments such as the differentiation of female employment and the differentiation of family patterns should be reflected in the measures.

Additionally, the present study reports the first year in which an item was introduced to see when it first became relevant (see the x-axes in Fig. 2). Furthermore, the number of years an item is used is subtracted from the mean number of years for the use of all items. Thus, this indicator can take negative or positive values (an item less or more widespread than the overall average) (see the y-axes in Fig. 2) and shows how relevant an item is compared to other items.

The different foci are evaluated separately for role ascription, role conflict, and role segregation. The results of the analysis are presented in Fig. 2.

Each mark in the Figure represents one item from Table 1. Regarding the first aspect role ascription (A) and role conflict (B), the analysis shows that most items refer to women (focus gender: triangles), and the items referring to men (focus gender: squares) are not common. However, since the 1990s, surveys have introduced some items regarding men, especially related to role ascription. These items refer mainly to the roles ascribed to the private sphere (see Table 1). In other words, in the private sphere, men have faced new challenges, especially regarding parenthood and housework, and so surveys have accounted for these developments, at least partly. However, the number of items referring to men and their dispersion in surveys indicate that most surveys need to extend their measures about male gender roles.

In terms of the egalitarian or traditional phrasing of items, we observed that—regarding role ascription—some items have an egalitarian (Fig. 2a, focus phrasing: light grey diamond) or both an egalitarian and a traditional phrasing (Fig. 2a, focus phrasing: dark grey diamond). That is, the items account for the roles that have arisen increasingly due to societal developments in the last decades, such as a higher female employment rate, a higher employment rate for mothers, and a rising number of single parents.

However, for items related to role conflict or role segregation, we only have a few egalitarian items (Fig. 2b, c, focus phrasing: light grey diamond), and therefore little adjustment to societal developments. In terms of the focus specification of the item, we found that some items related to role ascription specify the ages of children or the amount of employment (and also found an approximately equal number of items that do not) (Fig. 2a, focus specification: black diamond). Therefore, these items account partly for developments, such as a higher differentiation of female employment, especially regarding part-time work and the employment of mothers according to the age of their children. Items related to role conflict and role segregation are not usually specified in terms of workload, especially of mothers, or the ages of the children (Fig. 2b, c, focus specification: dark grey diamond).

In general, most measures about the ascription of gender roles are not widespread in surveys. More than half of these items are asked less often than the overall mean of the items. Items measuring the consequences of role conflicts or role segregation are slightly more widespread, but most items often are not asked about. That is, for many items, we have only a small number of rounds in which they were used in a survey or a small possibility of comparing answers across surveys.

All in all, our analysis found that measures often are too strongly focused on traditional roles. Societal developments have raised new questions about, for example, the evaluation of more egalitarian models of the division of labor in the family and public sphere that cannot be answered with these traditionally focused measures. In other words, items are missing that could help, for example, to evaluate the models that are more widespread in society today, such as part-time work for women and full-time work for men. Thus, a supplementation of the measures in use is necessary. Across all aspects of gender roles (Fig. 2a–c), our analysis of the focus specification of an item has shown that few items specify the number of working hours or the ages of children, which affects the validity of the measures in use. On the one hand, for example, with respect to the evaluation of the consequences of employment, the amount of employment matters. Full-time employment should be evaluated differently than part-time employment. However, so far, most surveys do not specify this aspect of employment, which would not be a problem if respondents always interpret the term *employment* the same across time. However, female employment has become more differentiated. Furthermore, employment for women per se probably has changed meaning, for example, due to changed family patterns and women’s lower financial security. Hence, the changes over time in personal experiences and the cultural context that influence the interpretation of a question about gender roles also have become more differentiated. This situation suggests that a specification of the amount of employment is necessary. On the other hand, the evaluation of the consequences of employment or the labor force division should also be dependent on the presence and the ages of children. In the 1970s, it was less common than today for a mother to participate in the labor force. Today, it is more common, but differences still exist according to the ages of children. Thus, today more than in the 1970s, the age of a child matters with respect to the evaluation of gender role attitudes. That is, since societal developments may have led to new interpretations of these terms, it is important to specify as many terms as possible to increase the probability that the measures are understood by every respondent in the same way. Furthermore, it helps to compare answers to questions across groups within a society and over time.

## Conclusion and discussion

Measures of gender role attitudes were mainly developed in the 1970s and 1980s when the male bread-winner model was dominant. However, societal developments, such as a greater differentiation of family structure and female employment led to an erosion of this model. This erosion also affects the measures of gender role attitudes. So far, most measures concentrate on traditional division of labor within the family, and so the evaluation of more egalitarian models is neglected. Furthermore, the role of men has changed, and some surveys already have adapted to these changes. However, especially regarding the evaluation of the consequences of employment, we do not have much information about how the male role is perceived. The differentiation particularly of female employment challenges the adequacy of items that are supposed to measure attitudes toward the division of labor and the consequences of employment, although these items usually do not specify the ages of children or the workload. To be able to compare answers, the room for interpretation should be small to ensure the equivalence of measures over time. Thus, a specification of these aspects of measurement is advisable. However, such a specification will directly affect the measures. We have to deliberate about whether we change some measures to ensure equivalence over time and risk the possibility of comparing current attitudes with attitudes measured in previous rounds. In summary, the question about whether measures of gender role attitudes are still adequate has to be answered in the negative. Of course, some measures are still useful for evaluating traditional models of the division of labor within the family, and some can even account for more egalitarian models and the newer roles of men or women in the public and private spheres. However, for future analyses of gender role attitudes over time, we still need to question the adequacy of the analyzed measures. Societal developments challenge the assumption that the measures developed in the 1970s measure the same concept or imply the same meaning as I have shown for gender role attitudes. Of course this problem affects not only measures of gender role attitudes, but also measures that try to capture developments over time. If we want to measure attitude change over time, we should always reflect critically on the adequacy of measures. Further steps regarding gender roles would be to evaluate whether some measures in use that already take some important developments into consideration can be adopted from other surveys as well. In general, a standard of measures towards gender role attitudes does not exist, which impedes analysis over time. It is necessary to develop new measures, especially concerning the role of men and more egalitarian models of the division of labor. Furthermore, concepts have to be developed concerning how measures in use can be adjusted to societal developments without risking important information about these developments over time. Therefore, tests also are necessary to see how changes in measures affect the responses to these changed measures. In addition, a specification of items is partly necessary, although this specificity also must be restricted to ensure the comprehensibility of an item. That is, it is a balancing act between specifying important aspects and keeping the new item as comprehensible as possible. The addition of new items regarding new aspects of gender roles also is restricted not only in terms of comparability over time, but also with respect to the time constraints of surveys. That is, the necessary adjustment of old measures of gender role attitudes is a challenging task that requires a consideration of many aspects. Finally, the measures in use were developed in a time in which sex-role theory was dominant, which was based on ideas of structural functionalism that saw gender roles as important to maintaining a well-functioning social system. Even though the ideas of structural functionalism were challenged early on, the idea of gender roles persisted and is still relevant for social psychology and survey methodology in particular. Thus, I discussed the revision of measures based on sex-role theory and did not take theoretical changes into consideration, although I am aware that several other theories have pointed out the limitations of sex role theory (e.g., Messner 1998; Risman and Davis 2013). Acker (1992), for example, has argued that gender is not limited to a social role, a personality component, or an individual attribute; in addition, gender also is a structural factor that is expressed by gendered institutions. Therefore, gender is present “in the processes, practices, images and ideologies, and distributions of power in the various sectors of social life” (p. 567). Ridgeway has emphasized that social interactions have played an important role in the maintenance of gender inequality (Ridgeway 2011; Ridgeway and Smith-Lovin 1999). We need new measures to test these theories that go beyond a gender role approach. A consideration of theoretical changes regarding gender theory would probably lead to an extension of measures related to gender in addition to measures of gender role attitudes in surveys. Future research therefore should address how theoretical changes would affect the adjustment and supplementation of measures regarding gender in surveys.
